# Characterization of carbapenem-resistant hypervirulent *Acinetobacter baumannii* strains isolated from hospitalized patients in the mid-south region of China

**DOI:** 10.1186/s12866-020-01957-7

**Published:** 2020-09-14

**Authors:** Jun Li, Ting Yu, Yi Luo, Jing-Yi Peng, Yu-Jia Li, Xiao-Yan Tao, Yong-Mei Hu, Hai-Chen Wang, Ming-Xiang Zou

**Affiliations:** Department of Clinical Laboratory, Xiangya Hospital, Central South University, Changsha, 410008 Hunan China

**Keywords:** *Acinetobacter baumannii*, Carbapenem resistance, Whole-genome sequencing, Hypervirulent, Biofilm

## Abstract

**Background:**

*Acinetobacter baumannii* has traditionally been considered an opportunistic pathogen with low virulence. In this study, we characterized the carbapenem-resistant hypervirulent *A. baumannii* (CR-hvAB) stains isolated from our hospital in mid-south region of China.

**Results:**

Blood samples collected between January 2017 and May 2019 were used for virulence experiments and biofilm assays of individual carbapenem-resistant *A. baumannii* (CR-AB) strains, performed using a *Galleria mellonella* infection model and crystal violet staining method, respectively. CR-AB isolates that induced high mortality in the *G. mellonella* infection model were subjected to genotyping, susceptibility testing, and clinical data analysis, and the genetic characterization of these isolates was performed by whole-genome sequencing (WGS). Among the 109 CR-AB clinical strains, the survival rate of *G. mellonella* larvae infected with 7 (6.4%) CR-AB isolates (number of strains with mortality of 0, 10 and 20% was 4, 1, and 2, respectively), was significantly lower than that of *A. baumannii* ATCC 19606 (100.0%) and the remaining CR-AB isolates (> 80.0%). Consistent with these results, patients infected with these seven isolates had an average 7-day mortality rate of 42.9%, suggesting that the isolates were CR-hvAB. These seven isolates belonged to four sequence types (STs): ST457, ST195, ST369, and ST2088 (a new ST), and mainly ST457 (*n* = 4). The results of the biofilm study showed that eight strains had powerful biofilm ability (strong [*n* = 1] and moderate [*n* = 7] biofilm producers) including these seven CR-hvAB isolates.

**Conclusions:**

CR-hvAB isolates that induced a high mortality rate were cloned in our hospital, most of which belonged to ST457; thus, monitoring of these strains, particularly ST457, should be strengthened in the future. Meanwhile, *A. baumannii*, which was isolated from blood specimens and found to powerful biofilm-forming ability, is a probable hvAB isolate.

## Background

*Acinetobacter baumannii* is a member of the ESKAPE pathogens, which includes six nosocomial bacteria (*Enterococcus faecium, Staphylococcus aureus, Klebsiella pneumoniae, Acinetobacter baumannii, Pseudomonas aeruginosa, Enterobacter spp*.), and causes infections and antimicrobial resistance in bacteria [1, 2]. Over the last decade, it has become notorious for its widespread drug resistance, such as carbapenem-resistant, extensively drug-resistant, and even pan drug-resistant *A*. *baumannii*, which severely threatens human health [3–7]. Due to increased antibiotic resistance, this bacterium was identified by the World Health Organization (WHO) as one of the world’s leading critical pathogens in 2017 [8].

*A. baumannii* is traditionally considered to be a low virulence pathogen causing opportunistic infection in immunodeficient individuals. However, it has evolved to be hypervirulent with high mortality and even clonal spread in specific hospitals [9–11]. In China, *A. baumannii* with enhanced virulence has been found not only in animals (chicks) [12] but also in humans in three regions (Guangdong, Zhejiang, and Hebei) [11, 13]. To date, it has not been reported in Hunan region.

Biofilm is an important structure of bacteria, mainly composed of extracellular mucopolysaccharide and other substrates secreted by bacteria to adapt to the changes of the surrounding environment [14]. In *A. baumannii*, biofilm formation is related to phenotypic variation, including physicochemical regulation and antibiotic resistance [15]. However, the role of biofilm formation in the evolution of hypervirulent of *A. baumannii* remains unclear. In fact, there is currently no clear definition for identifying hypervirulent isolates. In previous studies [9–11], hypervirulent isolates were defined according to their high mortality in an animal infection model; therefore, they were similarly defined in our study.

We investigated the epidemiology, clinical relevance, and genetic variations of carbapenem-resistant hypervirulent *A. baumannii* (CR-hvAB) isolates confirmed by the *Galleria mellonella* infection model collected from a hospital in the mid-south region of China. Meanwhile, the biofilm-forming capacity of CR-AB strains was tested by the microtiter plate method to analyze the correlation between biofilm and hypervirulence.

## Results

### Clinical characteristics of CR-hvAB

**Fig. 1:**
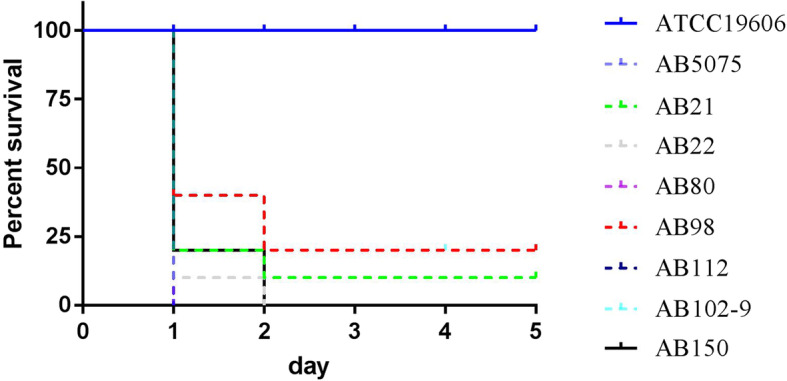
Virulence of seven CR-hvAB isolates. The impact of 1 × 10^6^ colony-forming units of each *A. baumannii* strain on survival were examined in *G. mellonella* infection model. Isolates AB21, AB22, AB80, AB98, AB112, AB102–9, and AB150 were assessed in this assay. ATCC 19606 and AB5075 were included as a low, and hypervirulent *A. baumannii* control. The survival rate of seven isolates were 20.0% (*n* = 2), 10.0% (*n* = 1), and 0% (*n* = 4), and significantly lower than that of ATCC 19606 (100.0%)

A total of 109 CR-AB isolates were collected. The survival rate of *G. mellonella* larvae infected with 7 (6.4%) CR-AB isolates (the number of strains with survival rates of 0, 10 and 20% was 4, 1, and 2, respectively, shown in Fig. 1) was significantly lower than that of *A. baumannii* ATCC 19606 (100.0%) and the remaining CR-AB isolates (> 80.0%). Consistent with these results, patients infected with these seven isolates (AB21, AB22, AB80, AB98, AB102–9, AB112, and AB150) had an average 7- and 21-day mortality rate of 42.9 and 71.4%, respectively, suggesting these seven isolates were CR-hvAB. As shown in Table 1, the clinical characteristics of these seven patients infected with CR-hvAB were different. Among them, six recovered in the intensive care unit and one recovered in the surgical department. Four of them had diabetes, while the others had no underlying diseases. In particular, AB102–9 was isolated from an adenovirus antigen-positive 19-year-old patient without any underlying diseases.

**Table 1:** Clinical characteristics of seven CR-hvAB isolates

Patient	Age-range	Isolate	Department	Date of isolation	Diagnosis	Underlying diseases	Outcome	MLST	Resistant phenotype
1	10–20	AB102–9	ICU	2019.5.14	Severe pneumonia	–	Recovered	ST457	TZP, CRO, CAZ, CFP, IPM, MEM, AK, GEN, CIP
2	20–30	AB21	ICU	2018.2.17	Severe pneumonia	DM	Death	ST369	TZP, CRO, CAZ, CFP, IPM, MEM, AK, GEN, CIP, TMP-SMZ
3	60–70	AB22	ICU	2018.3.9	Sepsis	DM	Death	ST457	TZP, CRO, CAZ, CFP, IPM, MEM, AK, GEN, CIP
4	30–40	AB80	SD	2018.1.11	Wound	–	Recovered	ST2088	TZP, CRO, CAZ, CFP, IPM, MEM, CIP, TMP-SMZ
5	60–70	AB98	ICU	2017.1.17	Sepsis	DM	Death	ST457	TZP, CRO, CAZ, CFP, IPM, MEM, AK, GEN, CIP
6	60–70	AB112	ICU	2017.4.15	Septic shock	DM	Death	ST195	TZP, CRO, CAZ, CFP, IPM, MEM, AK, GEN, CIP
7	60–70	AB150	ICU	2017.10.4	Septic shock	–	Death	ST457	TZP, CRO, CAZ, CFP, IPM, MEM, AK, GEN, CIP

*ICU* intensive care unit, *SD* surgical department, *DM* Diabetes; −, No underlying diseases, *TZP* piperacillin / tazobactam, *CRO* Ceftriaxone, *CAZ* ceftazidime, *IPM* imipenem, *MEM* meropenem, *GEN* gentamicin, *AK* amikacin, *CIP* ciprofloxacin, *CFP* cefepime, *TMP-SMZ* trimethoprim and sulphame-thoxazole

### Antimicrobial susceptibility test and resistance gene detection

**Fig. 2:**
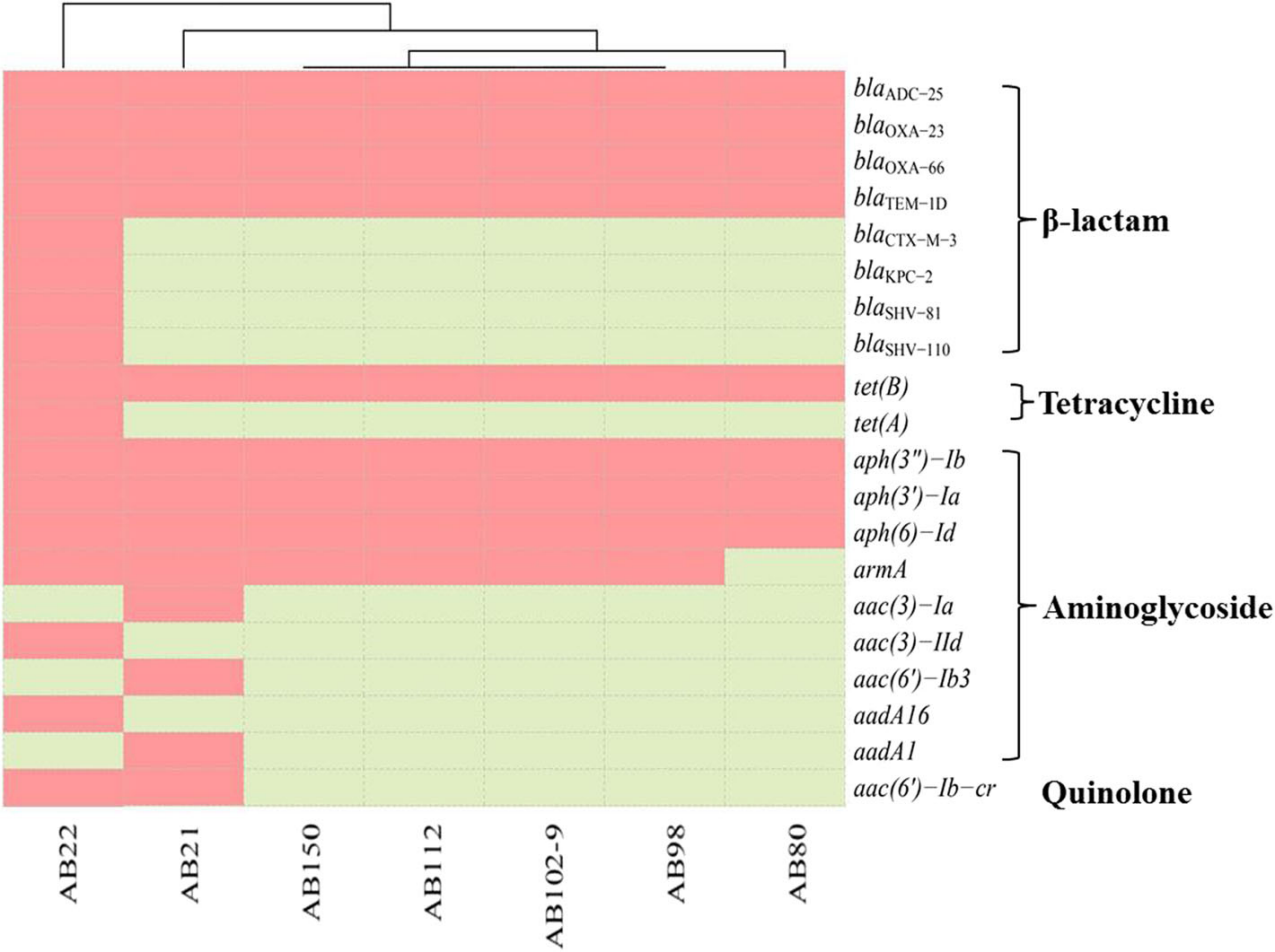
Four types of resistance genes were identified from seven CR-hvAB isolates collected in this study. The presence of genes is measured by scores of blast greater than 0.6. The red regions represent positive, and the blue regions represent negative

All CR-hvAB isolates were resistant to most common antibiotics except colistin and tigecycline (Table 1). The seven CR-hvAB isolates harbored multiple drug resistance genes, including *bla*_OXA-23_. It should be noted that AB22 is a co-carrier of both *bla*_OXA-23_ and *bla*_KPC-2_. Plasmid mediated *aac (6′)-lb-cr* gene was also detected in two CR-hvAB isolates (AB21 and AB22) (Fig. 2).

### Virulence genes

**Fig. 3:**
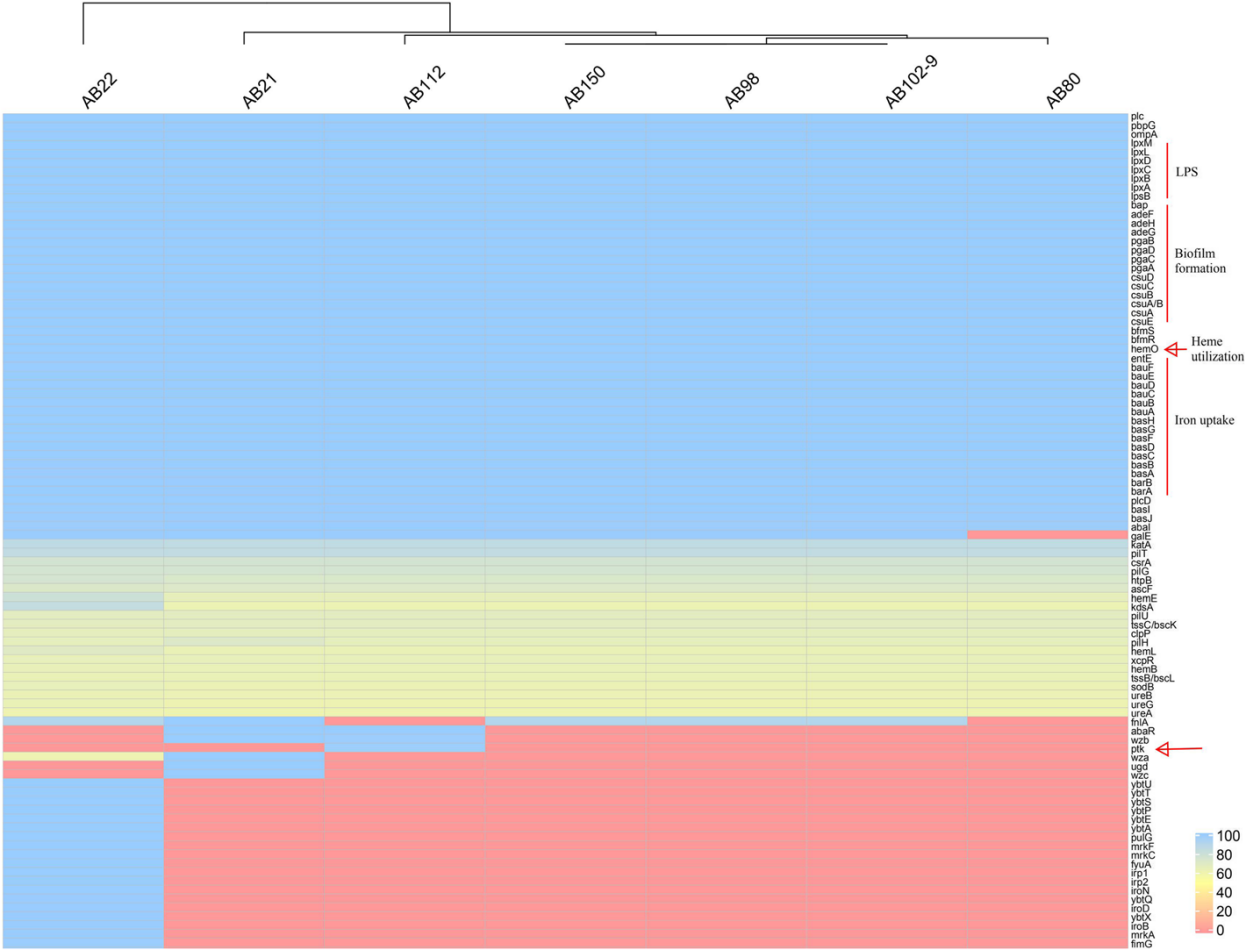
Identification of the virulence factors in seven CR-hvAB isolates. The presence of genes is measured by scores of blast greater than 0.6

As shown in Fig. 3, the virulence genes of AB98, AB102–9, and AB150 was almost identical, whereas those of the other four isolates were different. The *hemO* cluster, one of heme utilization gene clusters encoding for heme oxygenase, was found in all seven CR-hvAB isolates. Genes related to the uptake, acquisition, and transportation of iron such as *barA*, *barB*, *basC*, *basD*, and *bauA* were also found in all seven CR-hvAB isolates. Biofilm-associated genes including *bap* and *ompA* were also detected in each of the CR-hvAB isolates. In contrast, the *epsA* gene encoding a putative polysaccharide export outer membrane protein was not found in any of the seven CR-hvAB isolates [16]. The *ptk* gene, which encodes the putative protein tyrosine kinase involved in the synthesis of capsular polysaccharide [16], was only detected in AB112.

### MLST and phylogetic tree analysis

**Fig. 4:**
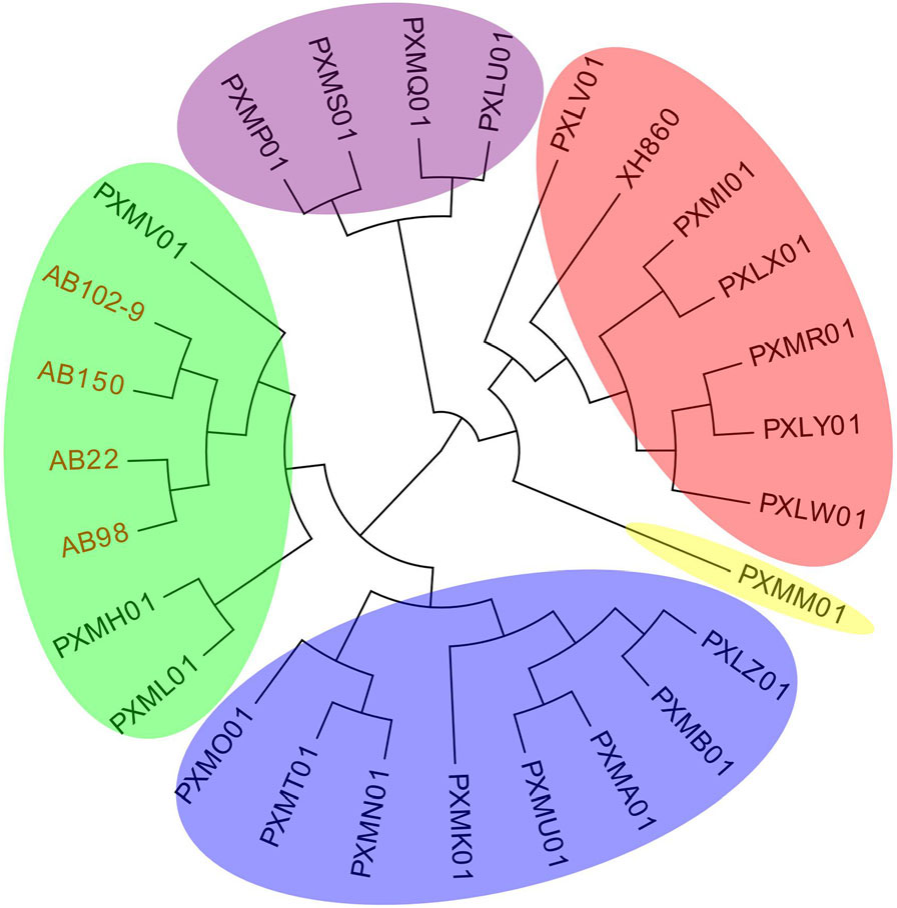
Phylogenetic tree of AB22, AB98, AB102–9, AB150, and ST457 hvAB published on NCBI based on SNP strategy was carried out by BacWGSTdb server with the reference isolate ST457 XH860 (CP014538). Different groups are represented by different colors

The seven isolates belonged to four STs including ST457 (AB102–9, AB22, AB98, and AB150), ST195 (AB112), ST369 (AB21), and ST2088 (1–35–3–2–2–221–3, a new ST) (AB80) (Table 1). The single nucleotide polymorphism (SNP) analysis of seven strains showed that they differed by 2176 SNPs, and the four ST457 CR-hvAB strains had a range of 0 to 12 SNPs. It should be noted that among the four ST457 CR-hvAB strains, AB98 and AB150 were isolated from 2017, while AB102–9 and AB22 were isolated from 2018 and 2019, respectively. To clarify the relationship between these four strains and ST457 hvAB published on NCBI, the SNP strategy-based phylogetic tree analysis was carried out on BacWGSTdb server using isolate ST457 XH860 (CP014538) as the reference. The results showed that 27 strains included in the analysis could be classified into five groups, of which AB102–9, AB22, AB98, AB150, PXMV01, PXMH01, and PXML01 belonged to the same group, while XH860 belonged to another group. In addition, the four ST457 hvAB strains isolated in this study are closely related to PXMV01, which was collected from a blood sample of a Guangzhou pneumonia patient in 2014 reported by Zhou et al. [11] (Fig. 4).

### Biofilm production assay

**Fig. 5:**
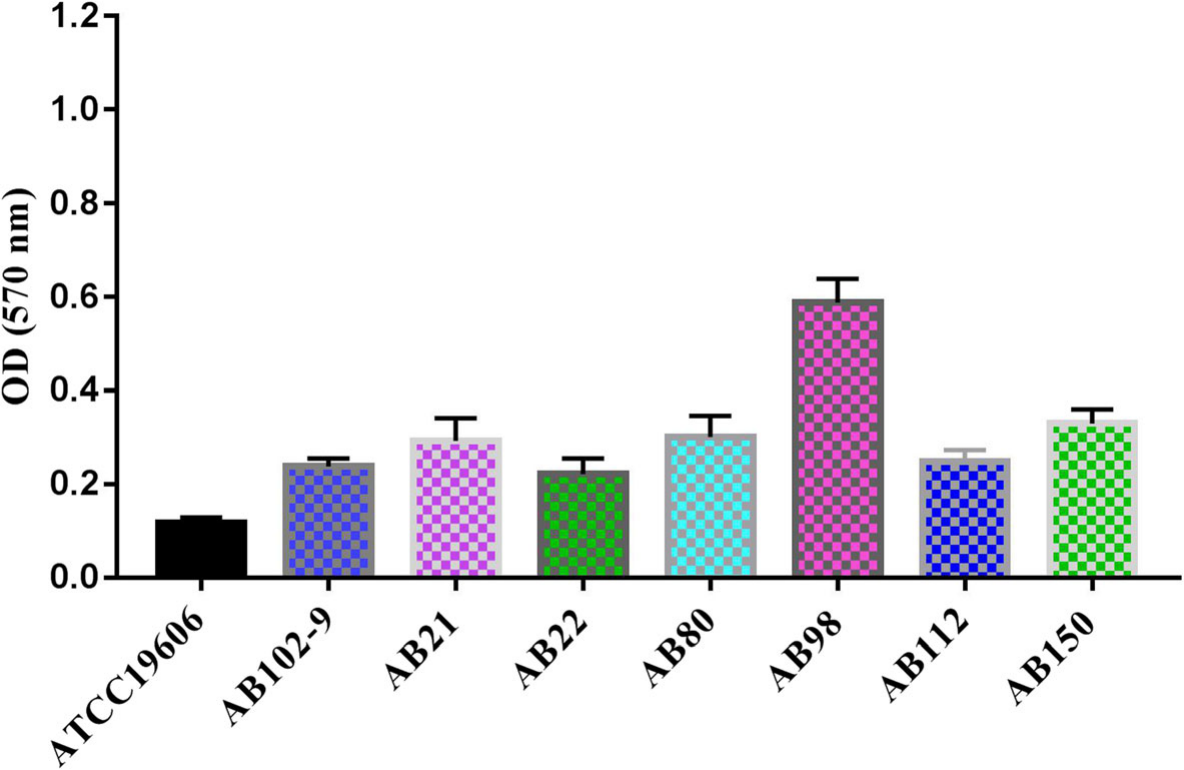
Biofilm formation of hvAB isolates was examined in this study. Biofilm formation of isolates AB21, AB22, AB80, AB98, AB112, AB102–9, and AB150 were examined. ATCC 19606 was included as a negative control

To investigate the relationship between the virulence and the biofilm-forming ability of isolates, the biofilm production assay of 109 isolates was conducted. The biofilm-forming ability of 109 isolates were classified into four categories: strong (*n* = 1), moderate (*n* = 7), weak (*n* = 96), and non-biofilm producer (*n* = 5). Of these eight isolates (one strong and seven moderate biofilm producers), seven were confirmed CR-hvAB isolates, as shown in Fig. 5. Only one non-hypervirulent *A. baumannii* isolates, AB66 was confirmed as a moderate biofilm producer.

## Discussion

In this study, we investigated the epidemiology, clinical significance, and genetic characterization of CR-hvAB from a hospital in the mid-south region of China. Meanwhile, the correlation between biofilm formation and virulence of CR-AB isolates was examined.

Of the 109 CR-AB isolates, 7 (6.4%) were proven to be CR-hvAB using the *G. mellonella* larvae infection model, which is widely used to evaluate the bacterial pathogenesis [11, 17, 18]. These seven strains were collected from patients with different clinical characteristics including a healthy young patient without any underlying diseases, suggesting that CR-hvAB isolates can cause infections in immunodeficiency and even immunocompetent patients [9]. The MLST showed that four of seven CR-hvAB isolates belongs to the ST457, which was initially isolated from Israel in 2013–2014 [19]. In China, ST457 has been identified in the south [11, 20] and southwest region [21], and increasing endemicity was first observed in Hong Kong in 2012–2014 [22]. Zhou et al. [11] reported that ST457 *A. baumannii* isolates were only second to ST195 and had a higher 7-day/28-day mortality rate than other STs of CC92, except for ST195 and ST208, and enhanced virulence with a high mortality rate through use of *G. mellonella* larvae infection model. In our study, three in four patients infected by ST457 CR-hvAB isolates died, and the 5-day survival rate of *G. mellonella* larvae infected with the four isolates were less than 20.0% (Fig. 1). Based on the above results, we speculate that ST457 has both broad resistance and enhanced virulence, and therefore has the potential for widespread transmission, at least in Asia. Therefore, the surveillance of these strains should be strengthened to control their outbreaks. In addition, Jones et al. [9] reported ST10 hvAB isolates have been detected in the Northwest United States. Similarly, one ST195 hvAB isolate, which is wide clonal spread in *A. baumannii* in China [2, 11, 23–25], one ST369 and one ST2088 (a new ST) CR-hvAB isolates were also detected in our study.

The phylogeny results showed that the SNPs of the four ST457 CR-hvAB isolates ranged from 0 to 12, suggesting that they could be clonal in our hospital. In order to clarify the relationship between our four ST457 hvAB strains and those published on NCBI, phylogenic tree based on SNPs strategy was carried out by BacWGSTdb server with the reference isolate ST457 XH860 (CP014538). The results showed that the four ST457 hvAB strains are closely related to PXMV01 collected from a pneumonia patient in Guangzhou [11], which is close to the region where we conducted this study. Due to the lack of PXMV01 isolates, we were unable to determine whether the prevalence of ST457 detected in our region was caused by inter-regional transmission. In addition, we found that our ST457 isolate had evolved compared to the 2009 isolate of XH860, and that the four isolates harbored numerous virulence factors, consistent with a study conducted by Zhou et al. [11].

The seven CR-hvAB isolates also harbored multiple drug resistance genes against the most commonly used antibiotics in the clinic, which made it difficult to treat. Notably, AB22 not only carried the *bla*_OXA-23_ gene, but also the *bla*_KPC-2_ gene, which is predominantly found in *Klebsiella pneumoniae* and clonally distributed in China [26–28]. To the best of our knowledge, only three cases of *A. baumannii* containing *bla*_KPC-2_ genes have been reported [29–31], making our study the first report on the detection of biofilm-producing *Klebsiella pneumoniae* carbapenemase-producing *A. baumannii* isolate in China.

Previous studies have revealed that *A. baumannii* expresses several virulence genes, such as iron acquisition and K1 capsular polysaccharides synthesis systems, which make it pathogenic to humans and animals [32–34]. In line with a previous report, the *hemO* cluster, one of heme utilization gene clusters encoding for heme oxygenase, was found in all seven CR-hvAB isolates in our study [10]. However, Ou et al. [10] reported that even if the gene is carried by *A. baumannii* isolates, but it may be no more virulent than ATCC 17978, ATCC 17961 or clinical isolate AYE in the mouse model of intranasal infection. Hence, the role of this gene in hvAB remains to be proved by further study. The genes related to uptake, acquisition, and transportation of iron such as *barA*, *barB*, *basC*, *basD*, and *bauA* were presented in all seven CR-hvAB isolates. Biofilm-associated genes including *bap* and *ompA* were also detected in all seven CR-hvAB isolates.

Except for sputum samples, AB strains isolated from urine or urinary catheters samples have been previously reported [35, 36]. Only 8 of 109 CR-AB strains in our study were proved to be strong or moderate biofilm producers, suggesting that it may be related to the nature of the *A. baumannii* clinical isolates [33]. Moreover, seven of these eight isolates were demonstrated to be CR-hvAB by *G. mellonella* larvae infection model. Thus, we speculated that most of the hvAB recovered from blood specimen is likely to be a strong or moderate biofilm producer.

## Conclusion

In our study, CR-hvAB isolates were strongly pathogenic in immunodeficient and even immunocompetent patients and were mainly ST457, so the monitoring of ST457 strains should be strengthened in the future. On the other hand, we found that most of the hvAB isolated from blood specimen has powerful biofilm-forming capabilities, but the specific mechanism needs further studies to be made clear.

## Methods

### Bacterial strains

Non-repetitive carbapenem-resistant *A. baumannii* (CR-AB) identified by the VITEK-2 system (bioMérieux, Marcyl’Etoile, France) were isolated from blood samples in the Xiangya Hospital of Central South University (Changsha, China) from January 2017 to May 2019. All isolates were identified at the species level using matrix-assisted laser desorption/ionization time-of-flight mass spectrometry (MALDI-TOF MS; Bruker Daltonics GmbH, Bremen, Germany) with *E. coli* ATCC 25922 (National Center for Clinical Laboratories, Beijing, China) used as the quality control strain, and PCR targeting the 16S–23S rRNA gene intergenic spacer region.

### Antimicrobial susceptibility testing

The minimal inhibitor concentrations (MICs) of piperacillin/tazobactam, imipenem, meropenem, ceftazidime, ceftriaxone, cefepime, ciprofloxacin, gentamicin, amikacin, and trimethoprim/sulfamethoxazole, and colistin in *A. baumannii* isolates were measured by the broth microdilution test. The susceptibility breakpoints were interpreted according to the Clinical and Laboratory Standards Institute (2019) [37]. The MIC of tigecycline was determined by the E-test (bioMérieux) and explained in accordance with the U.S. Food and Drug Administration breakpoint (resistant breakpoint 2 μg/mL). *E. coli* ATCC 25922 was used as the susceptibility control.

### Infection assays

The virulence of *A. baumannii* was examined in *G. mellonella* larvae (Tianjin Huiyude Biotech Company, Tianjin, China) infection model as previously described [11]. Briefly, overnight cultures of *A. baumannii* single colony were adjusted to 1 × 10^8^ colony forming unit/mL with phosphate-buffered saline. After being injected with 10 μL of the above bacterial suspension, the larvae were incubated at 37 °C in the dark and survival was monitored continuously for 5 days. AB5075 and *A. baumannii* ATCC 19606 were used as high and low virulence control strains, respectively. All tests were performed in triplicate.

### Whole-genome sequencing and data analysis

Whole-genome sequencing (WGS) was performed to detect resistant genes and virulence factors in CR-hvAB isolates. Approximately 10 μg DNA extracted by the DNeasy UltraClean Microbial Kit (QIAGEN, Hilden, Germany) for each strain was used to construct two Illumina paired-end libraries with average insertion lengths of 500 base pairs (bp) and 2000 bp. Libraries were sequenced using the Illumina GA IIx platform (Illumina Inc., San Diego, CA, USA). The following reads were removed from raw data: 1) reads of 5 bp ambiguous bases, 2) reads of 20 bp low-quality (≤ Q20) bases, 3) adapter contamination, and 4) duplicate reads. The final cleaned reads with about 100× genome coverage for each strain were assembled by SOAPdenovo v1.05. The resistant genes and virulence factors were identified using CARD (https://card.mcmaster.ca/) provided by the CGE server (https://cge.cbs.dtu.dk) and VFDB (http://www.mgc.ac.cn/VFs/main.htm), respectively. The phylogetic tree based on the SNP strategy and core genome multilocus sequence typing (cgMLST) were performed using the BacWGSTdb server as previously described [38]. MLST was performed using the Oxford scheme, and the sequence types (STs) were assigned using the MLST database (http://pubmlst.org/abaumannii). XH860 (CP014538), the first reported *A. baumannii* ST457 isolated in Guangdong, China, was used as the phylogenomic reference. The sequences of AB102–9, AB21, AB22, AB80, AB98, AB112, and AB150 were submitted to NCBI under BioProject PRJNA610947.

### Biofilm assay

Biofilm formation of CR-AB isolates was measured by the crystal violet staining method as previously described [32]. The absorbance at 570 nm of each triplicate assay was recorded as the mean ± standard deviation. *A. baumannii* ATCC 19606 was used as the negative control. In accordance with the criteria established by Stepannovic et al. [39], the cut-off value of optical density (ODc) was calculated using the following formula: ODc = average OD of the negative control + (3 × standard deviation of negative control). Based on the optical density results, the biofilm formation ability was divided into the four categories: (1) strong biofilm producer (OD > 4 × ODc); (2) medium biofilm producer (4 × ODc ≥ OD > 2 × ODc); (3) weak biofilm producer (2 × ODc ≥ OD > ODc); and (4) non-biofilm (OD ≤ ODc).

### Statistical analysis

Statistical analysis was conducted using SPSS19.0 software (SPSS Inc., Chicago, IL, USA). For the *G. mellonella* assays, survival curves were assessed using Kaplan-Meier analysis and the log-rank test. *P* < 0.05 was considered statistically significant.

## Data Availability

The datasets generated and analyzed during the present study are available from the corresponding author upon reasonable request.

## Abbreviations

CR-hvAB: Hypervirulent *Acinetobacter baumannii*
CR-AB: Carbapenem-resistant *Acinetobacter baumannii*
WGS: Whole-genome sequencing
STs: Sequence types
WHO: World Health Organization
Odc: Optical density
SD: Surgical department
DM: Diabetes
TZP: Piperacillin/tazobactam
CRO: Ceftriaxone
CAZ: Ceftazidime
IPM: Imipenem
MEM: Meropenem
GEN: Gentamicin
AK: Amikacin
CIP: Ciprofloxacin
CFP: Cefepime
TMP-SMZ: Trimethoprim and sulphame-thoxazole
